# Ploidy Variation in *Kluyveromyces marxianus* Separates Dairy and Non-dairy Isolates

**DOI:** 10.3389/fgene.2018.00094

**Published:** 2018-03-21

**Authors:** Raúl A. Ortiz-Merino, Javier A. Varela, Aisling Y. Coughlan, Hisashi Hoshida, Wendel B. da Silveira, Caroline Wilde, Niels G. A. Kuijpers, Jan-Maarten Geertman, Kenneth H. Wolfe, John P. Morrissey

**Affiliations:** ^1^School of Medicine, UCD Conway Institute, University College Dublin, Dublin, Ireland; ^2^School of Microbiology, Centre for Synthetic Biology and Biotechnology, Environmental Research Institute, APC Microbiome Institute, University College Cork, Cork, Ireland; ^3^Department of Applied Chemistry, Graduate School of Sciences and Technology for Innovation, Yamaguchi University, Yamaguchi, Japan; ^4^Department of Microbiology, Universidade Federal de Viçosa, Viçosa, Brazil; ^5^Lallemand Inc., Montreal, QC, Canada; ^6^Heineken Supply Chain, Zoeterwoude, Netherlands

**Keywords:** lactose transport, non-conventional yeast, yeast evolution, industrial yeast, dairy, Kluyveromyces, LAC12

## Abstract

*Kluyveromyces marxianus* is traditionally associated with fermented dairy products, but can also be isolated from diverse non-dairy environments. Because of thermotolerance, rapid growth and other traits, many different strains are being developed for food and industrial applications but there is, as yet, little understanding of the genetic diversity or population genetics of this species. *K. marxianus* shows a high level of phenotypic variation but the only phenotype that has been clearly linked to a genetic polymorphism is lactose utilisation, which is controlled by variation in the *LAC12* gene. The genomes of several strains have been sequenced in recent years and, in this study, we sequenced a further nine strains from different origins. Analysis of the Single Nucleotide Polymorphisms (SNPs) in 14 strains was carried out to examine genome structure and genetic diversity. SNP diversity in *K. marxianus* is relatively high, with up to 3% DNA sequence divergence between alleles. It was found that the isolates include haploid, diploid, and triploid strains, as shown by both SNP analysis and flow cytometry. Diploids and triploids contain long genomic tracts showing loss of heterozygosity (LOH). All six isolates from dairy environments were diploid or triploid, whereas 6 out 7 isolates from non-dairy environment were haploid. This also correlated with the presence of functional *LAC12* alleles only in dairy haplotypes. The diploids were hybrids between a non-dairy and a dairy haplotype, whereas triploids included three copies of a dairy haplotype.

## Introduction

The yeast *Kluyveromyces marxianus* is best-known because of its frequent association with traditional dairy products such as kefir and cheese (Lachance, 2011; Gethins et al., 2016; Coloretti et al., 2017). This association with fermented dairy beverages, a consequence of its capacity to use the milk sugar lactose as a carbon source, has led to inclusion of *K. marxianus* on GRAS (FDA) and QPS (EU) lists of safe micro-organism for use in foods (Lane and Morrissey, 2010; Ricci et al., 2017). The yeast is also regularly isolated from non-dairy environments (e.g., decaying fruit) and is part of the natural flora involved in production of *Agave*-based alcoholic beverages such as tequila and mezcal (Lappe-Oliveras et al., 2008; Verdugo Valdez et al., 2011). In the latter case, the production of enzymes that degrade plant fructans to simpler sugars (inulinases) undoubtedly contributes to its growth in this environment (Arrizon et al., 2012). The capacity of *K. marxianus* to utilise a broad array of sugars also creates potential for biotechnological applications (Fonseca et al., 2008; Lane and Morrissey, 2010), which is illustrated by the many studies exploring potential for bioethanol production from diverse substrates such as whey permeate, crop plants, and lignocellulosic biomass (Nonklang et al., 2009; Guimarães et al., 2010; Wu et al., 2016; Kobayashi et al., 2017). This yeast is used commercially for production of the flavour molecule 2-phenylethanol, and there is considerable interest in development of *K. marxianus* as a cell factory for production of other bioflavours (Morrissey et al., 2015). *K. marxianus* is also distinguished by thermotolerance (Lane et al., 2011), and the fastest reported growth rate of any eukaryote (Groeneveld et al., 2009). Recent years have seen increasing interest in new applications such as production of biomolecules (Hughes et al., 2017; Lin et al., 2017), biocatalysis (Oliveira et al., 2017; Wang et al., 2017) and heterologous protein production (Gombert et al., 2016; Lee et al., 2017).

One of the interesting aspects of the nascent development of *K. marxianus* as an important yeast for biotechnology is the wide variety of strains that are being used, both for research and for application. This contrasts with the traditional yeast, *Saccharomyces cerevisiae*, where, until recently, there was a very strong focus on a relatively narrow set of model strains. While giving access to the broad diversity that exists within any species, the non-reliance on model strains also creates challenges since findings with one isolate are not automatically transferrable to other isolates. This is illustrated well by studies that demonstrate wide variance in tolerance to different external stresses (Lane et al., 2011; Rocha et al., 2011). Indeed, it has emerged that even a trait such as lactose utilisation, long considered one of the defining characteristics of *K. marxianus*, is not universal, and many strains exhibit very poor growth on lactose, a phenotype that was shown to be due to polymorphisms in the *LAC12* gene, which encodes a permease responsible for transport of lactose into the cell (Varela et al., 2017). Although recent studies on sugar transport and physiology are starting to address the deficit (Fonseca et al., 2013; Signori et al., 2014; Beniwal et al., 2017; Dias et al., 2017; Diniz et al., 2017), it is true to say that a lot of the underlying knowledge about the biology of *K. marxianus* is based on inference of similarity with its sister species, *Kluyveromyces lactis*, which was developed as a model for studying lactose-positive yeasts since the 1960's (Fukuhara, 2006). The genome of *K. lactis* was sequenced more than a decade ago (Souciet et al., 2000), with a more recent functional reannotation and genome scale model that provides a deeper understanding of the core metabolism of this species (Dias et al., 2012, 2014). Notwithstanding the utility of a related species for comparison, the many metabolic and physiological differences between *K. lactis* and *K. marxianus* necessitate independent studies of *K. marxianus* to provide the comprehensive understanding of its genetics and physiology that will underpin future developments in fundamental biology and biotechnology.

Genomic and transcriptomic studies have started to shed light on *K. marxianus* and a growing number of genome sequences of *K. marxianus* strains are now available (Jeong et al., 2012; Silveira et al., 2014; Inokuma et al., 2015; Lertwattanasakul et al., 2015; Quarella et al., 2016). As yet, however, there has not been a systematic comparison of the sequenced *K. marxianus* genomes, nor a comparison to the single *K. lactis* genome that is in the public domain. In contrast to *K. lactis*, whose genome comprises 6 chromosomes, several studies have reported that *K. marxianus* has a full complement of 8 chromosomes, with many areas of local synteny between the species. There is strong conservation of the mating type locus (Lane et al., 2011) and thus *K. marxianus* could be expected to be capable of mating type switching and mating in a manner similar to *K. lactis* (Barsoum et al., 2010; Rajaei et al., 2014). Based on information to date, however, there does appear to be a fundamental difference in life-cycles. Studies of natural isolates of *K. lactis* suggest that this yeast is primarily a haploid (haplontic) species. Mating is induced by depletion of nitrogen or phosphate in the environment, and zygotes formed by mating usually sporulate immediately (although diploids can be maintained in the lab, e.g., by selection for auxotrophic markers) (Schaffrath and Breunig, 2000; Zonneveld and Steensma, 2003; Booth et al., 2010; Rodicio and Heinisch, 2013). In contrast, analysis of the mating-type locus of natural and culture collection *K. marxianus* isolates identified both haploid and diploid strains (Lane et al., 2011; Fasoli et al., 2016).

To put the phenotypic diversity of *K. marxianus* into context, it is important to characterise its genomic diversity and to assess the population structure of the species. There have been some pre-whole genome sequence studies that addressed this question using different methods. Pulsed-field gel electrophoresis studies suggested that there were variable numbers of chromosomes in *K. marxianus* strains (Belloch et al., 1998; Fasoli et al., 2015), a finding not in accordance with genome sequence data, which has consistently indicated 8 chromosomes. Mitochondrial DNA haplotypes and variation at some genomic loci was used to try to determine population structure in a collection from Italian cheeses (Fasoli et al., 2016). That particular study identified variations in population structure and proposed the occurrence of homozygous and heterozygous strains. A Multi Locus Sequence Typing (MLST) method was developed to further explore the diversity in that collection and in this case, the analysis was extended to other strains that are sequenced or available in culture collections (Tittarelli et al., 2018). MLST analysis did not identify distinct sub-populations but while the method was very diagnostic for strain identification, the surprisingly high level of heterozygosity in diploid strains reduced resolution to a level too low for population-type analysis. Analysis of population structure in diploid yeasts is challenging and, in many cases, has relied on SNPs identified in genome sequences derived from haploids or from completely homozygous diploids (made by self-mating of single spore derivatives) (Liti et al., 2009; Schacherer et al., 2009; Strope et al., 2015). In highly heterozygous species, this method may not generate an accurate view of the relationships among haplotypes or among strains.

In this study, we set out to explore the genomic diversity of *K. marxianus* by analysing whole-genome data from 14 strains isolated from different sources. Some of these strains had been previously sequenced and published, whereas others were sequenced for this study. To take heterozygosity into account, raw sequence reads were used to allow analysis of single nucleotide polymorphisms (SNPs) between strains. The results indicate a high degree of variation among isolates, in both ploidy and heterozygosity, and show a correlation between ploidy and environmental niche. Our work raises important questions about the life cycle of *K. marxianus*, and emphasizes the need to take ploidy and heterozygosity into account when considering using *K. marxianus* for biotechnological purposes.

## Materials and methods

### Yeast strains, growth, and phenotypic analysis

The 14 *K. marxianus* strains analysed in this study are listed in Table 1. Two strains (DMKU3-1042 and UFS-Y2791) were not available for phenotypic assessment but the remaining 12 strains were obtained from the sources indicated in Table 1 and were routinely cultured at 30°C in YPD medium (10 g/L yeast extract, 20 g/L bactopeptone, 20 g/L glucose). For lactose utilisation tests, yeast strains were first grown overnight in 5 mL minimal media (MM) supplemented with 2% glucose (Fonseca et al., 2007). Cells from the overnight cultures were harvested by centrifugation, washed twice with 5 mL of water and used to inoculate MM supplemented with 2% lactose to an OD_600_ of 0.1. These cultures were incubated for 15 h, when the final OD_600_ was determined. Lactose concentration was determined by HPLC at 0 and 15 h and used to calculate lactose consumption as previously described (Varela et al., 2017). Experiments were performed in triplicate with error bars showing standard deviation.

**Table 1:** Sources of *K. marxianus* strains and genomes analysed in this study.

**Strain**	**Synonym**	**Country**	**Sample source**	**Strain source**	**Reference for genome sequence**	**Source of Illumina FASTQ data**	**Accession numbers for Illumina data**
L01		Unknown	Dairy	Lallemand Inc.	This study	University College Dublin (K.H. Wolfe)	SRX3541360
L02		Unknown	Dairy	Lallemand Inc.	This study	University College Dublin (K.H. Wolfe)	SRX3541359
L03		Unknown	Dairy	Lallemand Inc.	This study	University College Dublin (K.H. Wolfe)	SRX3541362
L04		Unknown	Baking	Lallemand Inc.	This study	University College Dublin (K.H. Wolfe)	SRX3541361
L05		Unknown	Distillery	Lallemand Inc.	This study	University College Dublin (K.H. Wolfe)	SRX3541364
CBS397		Netherlands	Yoghurt	Westerdijk Institute, Netherlands	This study	University College Cork (J.P. Morrissey)	SRX3541363
NBRC0272		Unknown	Miso	Biological Resource Center, NITE (NBRC), Japan	This study	Yamaguchi University (H. Hoshida)	SRX3541366
NBRC0288	DSM4906	Unknown	Unknown	Biological Resource Center, NITE (NBRC), Japan	This study	Yamaguchi University (H. Hoshida)	SRX3541365
NBRC0617	ATCC8622	Denmark	Yoghurt	Biological Resource Center, NITE (NBRC), Japan	This study	Yamaguchi University (H. Hoshida)	SRX3541358
NBRC1777		Japan	Soil	Biological Resource Center, NITE (NBRC), Japan	Inokuma et al., 2015^*^	Yamaguchi University (H. Hoshida)	SRX3541357
CBS6556	KCTC17555, ATCC26548	Mexico	Pozol	Westerdijk Institute, Netherlands	Jeong et al., 2012	Yonsei University (J. F. Kim)	SRX3637961
UFV-3	CCT7735	Brazil	Dairy	Universidade Federal de Viçosa, Brazil	Silveira et al., 2014	BIOAGRO, Brazil (F. M. L. Passos)	SRX3637959
DMKU3-1042†		Thailand	Soil	Strain not obtained	Lertwattanasakul et al., 2015	Yamaguchi University (H. Hoshida)	SRX3541367
UFS-Y2791		South Africa	*Agave americana* juice	Strain not obtained	Schabort et al., 2016	Univ. of the Free State (D. T. W. P. Schabort)	SRX3637960

* *The reference genome sequence of NBRC1777 (Inokuma et al., 2015) was based on an assembly of Pacific Biosciences and Ion Torrent data but the SNP analysis in this study used newly-generated Illumina FASTQ data*.

† *Genome sequence obtained from a ura3 derivative generated by UV mutagenesis*.

### Flow cytometry

DNA content was determined by flow cytometry using SYTOX green (Thermo-Fisher) as previously described (Haase and Reed, 2002). Yeast strains were grown in YPD at 30°C with 200 rpm agitation in a New Brunswick Innova 40/40 R orbital shaker (Eppendorf, Hamburg, Germany). Cultures were harvested by centrifugation and resuspended in 1 mL sterile water. Cells were then washed, resuspended in 400 μL sterile water and fixed by adding 950 μL 100% ethanol. The suspensions were incubated overnight at 4°C, then centrifuged and washed in 50 mM sodium citrate (pH 7.2). The cells were resuspended in 500 μL RNAse A solution (0.25 mg/mL RNAse A, 50 mM sodium citrate pH 7.2) and incubated for 1 h at 37°C. Then, 100 μL of 20 mg/mL Proteinase K was added to each sample and the tubes were incubated at 50°C for 2 h. Finally, 500 μL of SYTOX Green solution (4 μM SYTOX Green, 50 mM sodium citrate pH 7.2) was added to each tube. Samples were analysed using a BD FACSCelesta system (BD Biosciences, CA, USA) and the data was processed using FlowJo software v10 (BD Biosciences, CA, USA).

### Genome data, sequencing, and read mapping

The genomes of five strains (NBRC1777, CBS6556, UFV-3, DMKU3-1042, and UFS-Y2791) had previously been published and some of the authors kindly made the source Illumina FASTQ data available for this analysis. The 11 strains sequenced in this study are indicated in Table 1, accession numbers are provided for all strains and references are given when applicable. All strains were sequenced on Illumina HiSeq 2000 or 2500 instruments after Truseq genomic library preparation. Details of the sequencing data type and coverage for all 14 strains, including those sequenced elsewhere, are summarised in Table 2. We performed quality control checks for all libraries with FastQC v. 0.10.1 (https://www.bioinformatics.babraham.ac.uk/projects/fastqc/.) Reads for strain CBS6556 were trimmed using skewer v. 0.2.2 (Jiang et al., 2014) with parameters -q 20 -m pe -l 70. For BLAST analyses, *de novo* assemblies of each newly sequenced genome were made using SPAdes 3.5.0 (Bankevich et al., 2012).

**Table 2:** Summary of Illumina sequencing strategies and coverage for 14 strains used in SNP analysis.

**Strain**	**Mean coverage (x)**	Pairedness^*^	**Read length**	**Million reads**	**BWA algorithm**
L01	47	SE	50	16.6	aln samse
L02	47	SE	50	16.7	aln samse
L03	45	SE	50	16.7	aln samse
L04	45	SE	50	15.6	aln samse
L05	47	SE	50	16.2	aln samse
CBS397	141	PE	126	15.1	mem
NBRC0272	160	PE	100	20.4	mem
NBRC0288	212	PE	100	25.6	mem
NBRC0617	35	SE	50	11.6	aln samse
NBRC1777	110	PE	100	12.8	mem
CBS6556	623	PE	70-150	56.9	mem
UFV-3	359	PE	90	50.3	mem
DMKU3-1042	341	PE	100	44.6	mem
UFS-Y2791	50	PE	75-100	17.1	mem

* *SE, single-end; PE, paired-end*.

The NBRC1777 genome sequence was selected as a reference because of the high quality of its assembly into 8 chromosomes, and because our analysis confirmed the haploid nature of this strain (Inokuma et al., 2015). Sequencing libraries from all strains were aligned to the NBRC1777 reference using the Burrows-Wheeler Aligner (BWA) v. 0.7.9a-r786 (Li and Durbin, 2009) with default parameters. The BWA “mem” alignment algorithm was used for libraries with read length ≥100 bp, the “aln” alignment algorithm for libraries with read length <100 bp, and the alignment modes were set to “samse” and “sampe” for single-end and paired-end data respectively. Samtools v. 0.1.19-44428cd (Li et al., 2009) was used to remove unmapped reads from the BWA output files and to generate indexes for downstream steps. Picard tools v. 2.0.1 (http://broadinstitute.github.io/picard) function AddOrReplaceReadGroups was used to add identifiers to the BAM files, followed by MarkDuplicates to mark and discard PCR duplicates. Indel realignment and coverage calculation were performed using the RealignerTargetCreator, IndelRealigner, and DepthOfCoverage tools from the Genome Analysis Tool Kit (GATK) v. 3.5-0-g36282e4 (Van der Auwera et al., 2013). Mean coverage was calculated omitting a 19 kb region on chromosome 5 that contains the array encoding the rRNA genes. Coverage plots were obtained by calculating the average in 10-kb windows. Segment means were calculated using the R Bioconductor package DNAcopy v 1.50.1 (DOI: 10.18129/B9.bioc.DNAcopy).

### Variant calling

“Variable sites” were defined as the set of sites in the genome that contain a non-reference base, hereafter called “variants,” in at least one of the 14 strains. Variant calling in the 14 strains was done using the GATK tool HaplotypeCaller in DISCOVERY and GVCF modes, requiring a minimum quality score of 20. The output files were then used for multi-sample analysis using the GenotypeGVCF tool and a custom Perl script was used to remove all variants that had low genotype quality (GQ < 20), or had low approximate read depth (below 10% of the mean coverage for the sample excluding the rDNA locus and telomeric regions). For every remaining variable site, the output from GATK enabled us to calculate the empirical allele frequencies of the reference base (designated *fA*) and the variant (designated *fB* and referred as alternative allele frequencies), which sum to 1. Empirical allele frequencies were calculated at each variable site on each strain by dividing the allelic depth of the variant by the approximate read depth observed at that site (AD and DP fields in GATK's HaplotypeCaller). Each variant remaining after the filtering steps and having an *fB* > 0.15 is considered to be SNPs. Accordingly, variable sites were only used for further analysis when having a SNP. If *fA* ≥ 0.85 the strain was called homozygous for the reference base (*AA*) at this variable site, and if *fB* ≥ 0.85 it was called homozygous for the alternative base (*BB*). Sites with intermediate allele frequencies (0.15 < *fA* < 0.85) were called heterozygous (*AB*). Using these thresholds, a small number of SNPs were called in some strains later shown to be haploid. These apparent SNPs clustered in sub-telomeric regions known to contain repetitive DNA and are likely to be technical artefacts due to misaligning of reads to different repeats or to copy number variants. Note that these calls were only made for variable sites; the genomes also contain a much larger number of invariant sites that are considered identical among all strains and are therefore *AA*.

Nucleotide diversity (π) and average SNP density were calculated with VariScan v 2.0.3 (Hutter et al., 2006) using a non-overlapping window size of 1 kb along all chromosomes. In the case of heterozygous variants, only the variant with highest *fB* was used. Sites considered for the analysis of the 14 strains were required to show variation in a minimum number of 4 strains. SnpEff v 4.3s (Cingolani et al., 2012) was used to produce summary statistics and annotate the SNPs using the public NBRC1777 genome annotation as a reference (Inokuma et al., 2015).

### Phylogenetic analysis

Because the data consisted of a mixture of strains with different ploidies, we developed a custom method for phylogenetic analysis of haplotypes. This method is based on a window approach similar to our previous development for an interspecies hybrid (Schröder et al., 2016). Homozygosity and heterozygosity were first assessed in 1 kb windows of the genome of each diploid strain. For each 1 kb window in each strain, the total numbers of variable sites that were called with each genotype (#*AA*, #*BB*, and #*AB*) in the window were calculated. The whole window was then classified as either heterozygous for the two haplotypes if *#AB* ≥ 3, or homozygous otherwise. Homozygous windows were then classified as either homozygous for the alternative haplotype if *#BB* ≥ 9, or homozygous for the reference haplotype otherwise. The cut-off values of 3 and 9 were chosen based on analysis of the distributions of window frequencies, using strain L01 as a test case (Figure S1). Each 1 kb window of the genome was only used if it was heterozygous in all five diploid strains, and the regions with aberrant allele frequencies in NBRC0272 were excluded (chromosome 6). Concatenated nucleotide sequences of the shared heterozygous windows were extracted from the NBRC1777 genome and used to infer the sequences of alleles in each strain, depending on its ploidy.

For haploid strains, the variant was used to replace the reference base at the corresponding position of the variable site in the concatenated sequence. For diploid strains, two different putative A and B alleles were first generated and used as a template for base replacement depending on the type of variable site. For homozygous alternative (*BB*) sites, the variant was used for replacement in both A and B alleles. For heterozygous (*AB*) variable sites, the variant was only used for replacement in the B allele. For triploid strains, putative alleles 1, 2, and 3 were first generated and used as a template for base replacement depending on the types of variable sites and their allele frequencies. For homozygous alternative (*BB*) sites, the variant was used for replacement in all three putative alleles. For heterozygous (*AB*) sites, the variant was used for replacement in both alleles 2 and 3 if *fB* > 0.6, or for replacement only in allele 3 if *fB* < 0.4. The phylogenetic tree of the inferred and concatenated haplotype sequences was generated using PhyML v. 3.1 (Guindon et al., 2010) selecting for the best of NNI and SPR methods, using five random starts, and with empirical estimation of base frequencies and proportions of invariable sites (parameters: –search BEST –rand_start –n_rand_starts 5 -f e –v e). The tree was visualized using FigTree v. 1.4.3 (http://tree.bio.ed.ac.uk/software/figtree/.)

### Data availability

The Illumina sequences generated and analyzed for this study can be found in the NCBI Sequence Read Archive under the accession number SRP128575, strain-specific accessions are provided in Table 1.

## Results

### *K. marxianus* displays a high level of genomic variation

The genome sequences of 14 strains of *K. marxianus* were analysed for SNPs to determine the extent of variability in this species. The strains selected for analysis included the five strains with published whole genome sequences (at the time of the study) and 9 other strains from different collections (Table 1). The five sequenced strains (NBRC1777, CBS6556, UFV3, DMKU3-1042, and UFS-Y2791) have been phenotypically analysed to different extents by a number of research teams and are of interest for biotechnological applications (Nonklang et al., 2008; Fonseca et al., 2013; Costa et al., 2014; Schabort et al., 2016; Nambu-Nishida et al., 2017). This is also the case for the previously unsequenced strains CBS397, NBRC0272, NBRC0288, and NBRC0617, which were obtained from national culture collections (Lane et al., 2011; Foukis et al., 2012; Yarimizu et al., 2013). The additional five strains (L01–L05) are from the in-house culture collection of the Lallemand company and there are no published data available for these strains. The original source of isolation is known for 13/14 strains and is almost evenly divided between dairy and non-dairy environments. The genome sequences of the 9 previously un-sequenced strains were obtained as described in methods and thus all 14 genomes could be analysed and compared. It should be noted that although all 14 strains were sequenced using Illumina technology, this was performed by different laboratories using a diversity of sequencing strategies and thus the depth of coverage is quite variable (Table 2).

We used the genome sequence of strain NBRC1777, which was assembled into eight complete chromosomes using Pacific Biosciences technology, as the reference for SNP analysis (Inokuma et al., 2015). GATK software was used to identify sequence variants present in the Illumina reads from all 14 strains relative to this reference. Variable sites were defined as the set of sites in the genome that contain a non-reference base in at least one of the 14 strains. For each variable site in each strain, the empirical allele frequency in the Illumina reads from that strain was calculated (see Methods). Depending on frequency, the variant was classified as homozygous SNP (*fB* ≥ 0.85), or heterozygous SNP (0.15 < *fB* < 0.85). Variants appearing at frequencies below 0.15 were assumed to be due to sequencing errors and were ignored. Only 249 SNPs were identified in the Illumina data from the reference NBRC1777, confirming that the reference sequence is accurate and this strain is haploid. All other strains contained more than 30,000 SNPs relative to the reference (Table 3). The highest number of SNPs is in strain UFS-Y2791 (Schabort et al., 2016) and corresponds to 3.0% nucleotide sequence divergence in the 10.9 Mb genome. From the numbers of heterozygous and homozygous (non-reference) SNPs, the other strains fall into three groups: one group with low numbers of heterozygous SNPs (<2000), one group with more heterozygous than homozygous SNPs, and one group with fewer heterozygous than homozygous SNPs. Below, we show that these three groups are haploid, diploid, and triploid strains, respectively.

**Table 3:** SNPs identified in 14 *K. marxianus strains*.

**Strain**	Heterozygous SNPs^*^	**Homozygous SNPs^*†*^**	**Total SNPs**
**HAPLOID STRAINS**			
NBRC1777	248	1	249
L05	611	33,326	33,937
L04	726	35,138	35,864
CBS6556	1,439	40,922	42,361
DMKU3-1042	1,647	39,648	41,295
UFS-Y2791	714	3,25,190	3,25,904
**DIPLOID STRAINS**			
L02	1,15,648	42,561	1,58,209
L01	96,202	49,325	1,45,527
CBS397	1,10,132	52,415	1,62,547
NBRC0288	96,304	60,554	1,56,858
NBRC0272	1,47,241	40,085	1,87,326
**TRIPLOID STRAINS**			
NBRC0617	29,010	1,45,016	1,74,026
L03	26,388	1,45,856	1,72,244
UFV-3	27,347	1,77,365	2,04,712

* *Number of sites at which a variant (non-reference base) was present, at a frequency in the reads between 0.15 and 0.85*.

† *Number of sites at which a variant (non-reference base) was present, at a frequency ≥ 0.85 in the reads*.

In total, 667,472 variable sites, comprising 597,466 SNPs, and 70,006 indels were found. Indels were not analysed further, and, after filtering (see methods) a subset of 571,339 SNPs was retained for analysis. SNP diversity in *K. marxianus* is relatively high, with average pairwise difference between strains (π) of 12 × 10^−3^. The average density of SNPs in *K. marxianus* is 7.6 SNPs/kb in coding regions and 11.1 SNPs/kb in intergenic regions (Table S1). Of the 359,354 variants located within the coding regions of genes, 71.6% were predicted to be silent, 28.1% missense, and 0.2% (745) nonsense mutations, when compared against the NBRC1777 annotation (Inokuma et al., 2015).

### *K. marxianus* isolates show different ploidy states

**Figure 1:**
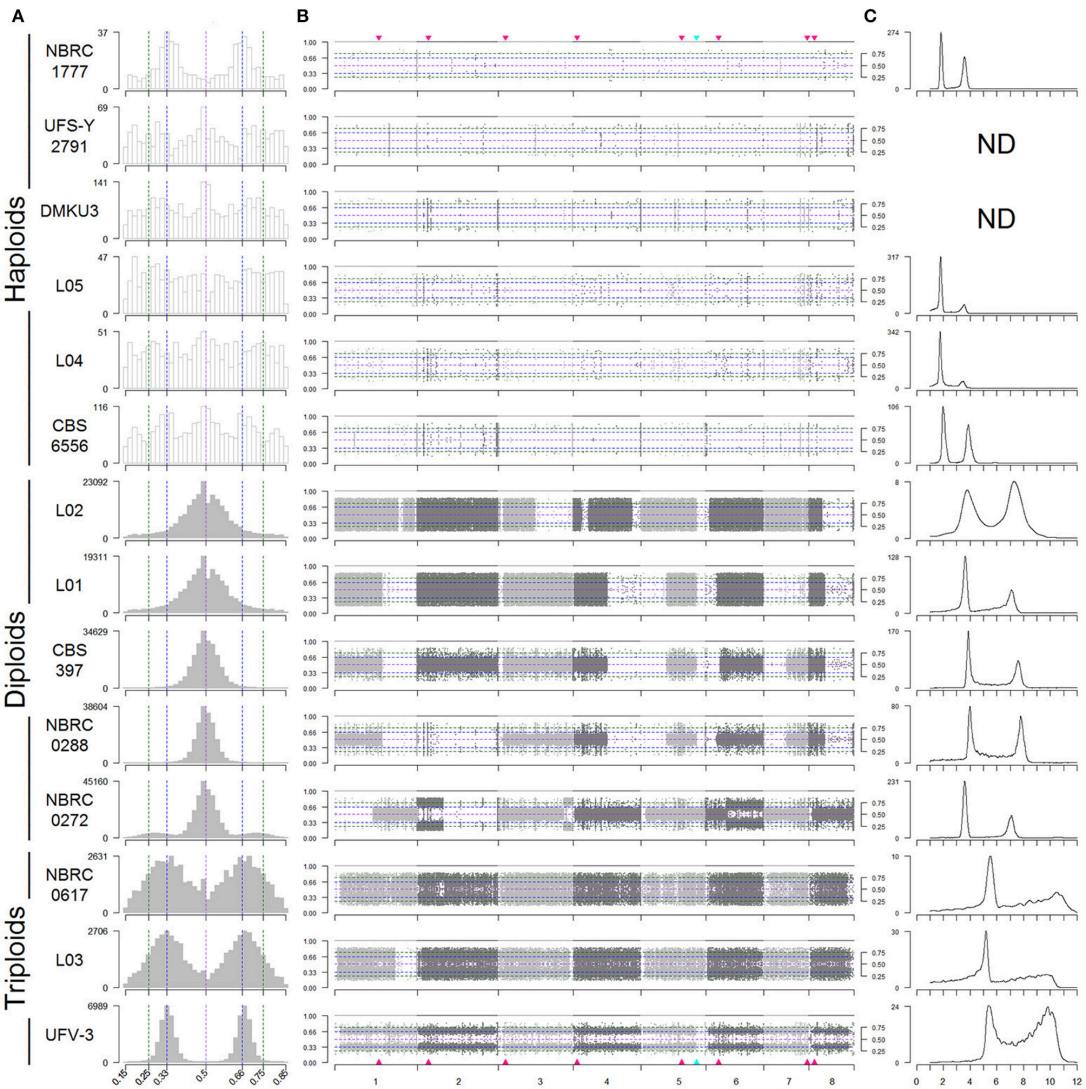
Variable ploidy in *Kluyveromyces marxianus* strains. Strain names are shown on the left. **(A)** Histograms of the alternative allele frequencies of variant (non-reference) bases, for SNPs designated as heterozygous (sites with alternative allele frequencies *fB* between 0.15 and 0.85). Histograms are coloured grey if at least 10% of the SNPs in a strain are heterozygous. Dashed vertical lines mark frequencies of 0.5 (purple), 0.33/0.66 (blue), and 0.25/0.75 (green). Bin sizes are 2% intervals. **(B)** Plots of alternative allele frequencies along the 8 chromosomes, for each strain. Horizontal dashed lines mark frequencies as in **(A)**. Light and dark gray points indicate SNPs on different chromosomes. Red triangles mark the locations of centromeres, and the blue triangle marks the ribosomal DNA locus. Allele frequencies ≥ 0.85 are shown as 1. Alternative allele frequencies ≤ 0.15 are not shown. **(C)** Flow cytometry of DNA content. The Y-axis shows numbers of cells, and the X-axis shows SYTOX Green fluorescence signal intensity (arbitrary units) which is proportional to DNA content. Flow cytometry was not carried out for UFS-Y2791 and DMKU3-1042.

The distribution of allele frequencies for each strain was assessed using a graphical method similar to that used in a recent study in *S. cerevisiae* (Zhu et al., 2016). Histograms (Figure 1A), of the distribution of allele frequencies at all the variable sites in the genome created three sets of strains that corresponded to those identified on the basis of the numbers of heterozygous and homozygous SNPs (Table 3). The five strains with the highest numbers of heterozygous SNPs in (L02, L01, CBS397, NBRC0288, and NBRC0272) all show a symmetrical peak of allele frequencies centred on 0.5, suggesting that they are diploid. The three strains with high numbers of both homozygous and heterozygous SNPs (NBRC0617, L03, and UFV-3) all show bimodal distributions, with peaks at 0.33 and 0.66 for the frequency of the variant, suggesting that they are triploid. In contrast, in the six strains UFS-Y2791, DMKU3-1042, L05, L04, CBS6556, and NBRC1777, only a low number of sites were designated as heterozygous, and these sites show little pattern in their frequency distributions. These data are most consistent with a haploid genome.

To provide an independent measurement of ploidy, DNA content in each strain was measured by flow cytometry (Figure 1C). Each analysed strain shows a bimodal distribution of DNA content, corresponding to the G1 and G2 phases of the cell cycle. For the haploid strains, the DNA content is 1*n* (in G1 phase) and 2*n* (in G2 phase), where *n* is the DNA content of the haploid genome. For the diploids, it is 2*n* and 4*n*, and for the triploids it is 3*n* and 6*n*. The patterns observed were consistent with the designation based on allele frequencies and confirmed that this set of strains was comprised of 6 haploid, 5 diploid and 3 triploid strains.

### Common patterns of loss of heterozygosity in diploid strains

For each SNP, its allele frequency vs. its chromosomal location in the reference assembly was plotted to determine whether heterozygosity was uniformly distributed (Figure 1B). For haploid strains, the low level of variation does not show any particular pattern, whereas in all five diploid strains, large regions of the genome with loss of heterozygosity (LOH) are apparent. In heterozygous regions of the genome, allele frequency should be distributed about 0.5 but there are regions where no variability is seen. These predominantly white areas in the plots indicate stretches of chromosome that are homozygous in that strain (Figure 1B). For example, strain L02 is heterozygous through most of its genome but shows homozygosity on the right half of chromosome 3 and over most of chromosome 8. In the diploids, most chromosomes are heterozygous over at least some of their length, but chromosome 2 in NBRC0288 and chromosome 7 in L01 are essentially completely homozygous. Three diploid strains L01, CBS397 and NBRC0288 exhibit almost identical extents of partial LOH on chromosomes 1, 3 (left end), 4, 5, 6, and 8 (Figure 1B), but different patterns on chromosomes 2 and 7. On chromosome 6, the region of LOH is slightly larger in CBS397 than in L01 and NBRC0288 (it crosses the centromere only in CBS397). Strain L02 shares two LOH boundaries with this group of three strains, on chromosome 5 (the boundary occurs at the rDNA locus) and chromosome 3 (left end). Only one region of LOH is visible in our triploids, on chromosome 1 in strain L03. This LOH region occupies ~4% of the L03 genome, whereas in the diploid strains the LOH regions total 25–51% of the genome by length.

### Copy number variation and partial aneuploidy in multiple strains

**Figure 2:**
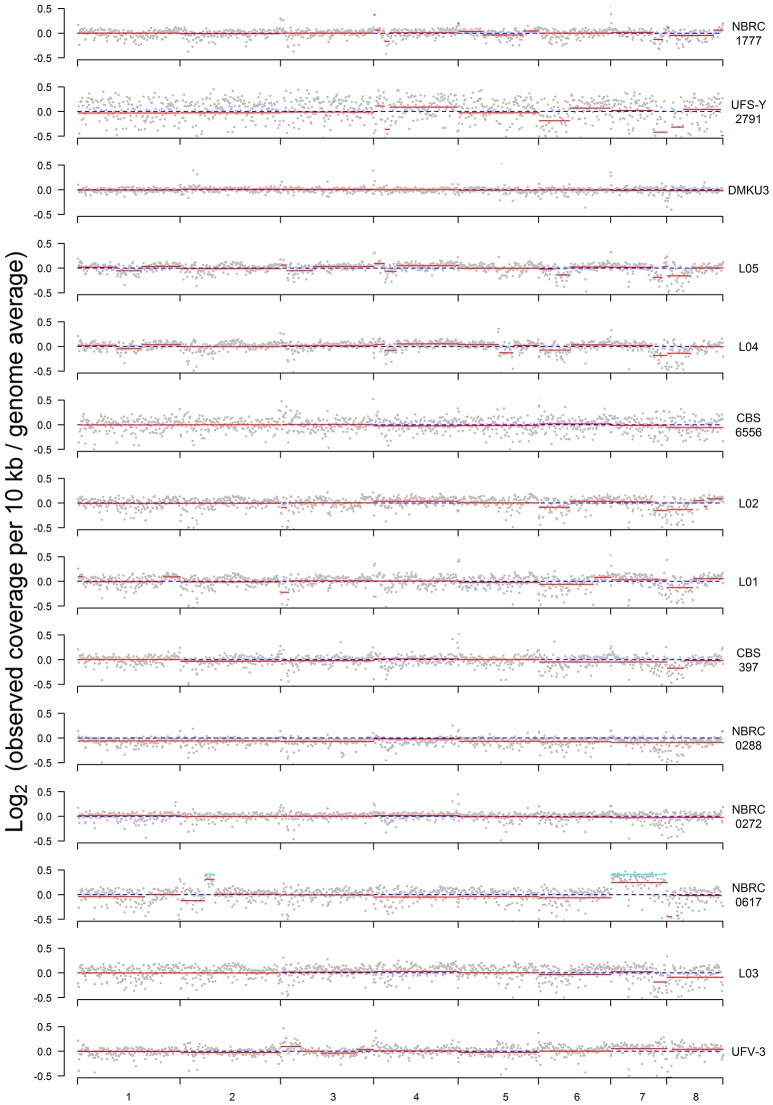
Plots of sequence coverage in each strain. The Y-axis is log_2_ of the ratio between the observed and expected coverage, for 10-kb windows through the genome; a value of zero (dashed blue line) indicates no difference. Expected coverage is based on the average in the whole genome. Red lines show the segmental means for consecutive 10-kb windows calculated using the Bioconductor package DNAcopy. Cyan lines for NBRC0617 indicate the value expected for the 1.33-fold increase in coverage that would result from a fourth copy of a region in a triploid.

Read coverage in 10 kb windows was determined across the genome of each strain to investigate whether any of the strains displayed aneuploidy (Figure 2). In this analysis, a value of zero indicates no variation between expected and actual numbers of reads, whereas higher or lower numbers could indicate DNA duplications or deletions. Although the SNP and flow cytometry results (Figure 1) indicated that there are three groups of strains with genomes that are primarily haploid, diploid, and triploid, there is also evidence in multiple strains of partial aneuploidy, segmental duplications, or deletions that alter the copy number of some parts of the genome. These possible aneuploidies do not correspond to the regions of LOH described in Figure 1, indicating that these are distinct phenomena.

The clearest case of aneuploidy is in strain NBRC0617, which has an extra copy of chromosome 7 (Figure 2). The analysis of ratios shows that reads from chromosome 7 are present at 1.18x the expected frequency (log_2_ value 0.249) in this strain. Since NBRC0617 is primarily triploid, a fourth copy of a chromosome should increase the read coverage on that chromosome by ~4/3 = 1.33-fold (cyan line in Figure 2) relative to the genome average, and many of the 10-kb windows in chromosome 7 are not significantly different from this value (Figure S2A). Furthermore, the distribution of allele frequency values for SNPs on chromosome 7 of NBRC0617 shows a peak at 0.25 (Figure S2B), which is consistent with the presence of four copies of the chromosome, and contrasts with the peaks at 0.33 and 0.66 that are seen when the whole genome of NBRC0617 is considered (Figure 1A). NBRC0617 also shows increased copy number of a circa 150 kb segment of chromosome 2 (coordinates 420–570 kb) (Figure 2). Within this segment, allele frequencies of 0.25 and 0.75 are visible (Figure S2B), indicating that it is present in 4 copies. With the current data, we are unable to determine the precise structure of the chromosomal rearrangement that increased the segment's copy number. The region immediately to the left of it (0–420 kb) may be present at a reduced copy number.

Some intriguing possible examples of copy number variation are seen in strain NBRC0272, which was designated as a diploid based on its overall allele frequency and flow cytometry patterns. Examination of allele frequencies indicates that there are three genomic regions (in chromosomes 2, 3, and 6) present at higher copy numbers (Figure 1B). This can be seen in more detail in the allele frequency plots of those chromosomes for this strain (Figure S3). Chromosomes 2 (left end) and 3 (right end) contain SNPs with 0.25/0.75 allele frequencies, indicating the presence of four copies. Chromosome 6 (right end) contains SNPs with allele frequency peaks close to 0.33/0.66, indicating three copies, and contrasting with the peaks at 0.5 on the left part of this chromosome. Since the flow cytometry (Figure 1C) shows that the total DNA content of NBRC0272 is close to diploid, it is concluded that the extra copies of these three chromosomal regions must be the result of segmental duplications and not extra copies of the whole chromosome. Puzzlingly, however, these putative segmental duplications were not apparent in the coverage plot of NBRC0272 (Figure 2).

### Phylogenetic analysis separates dairy from non-dairy haplotypes

The presence of strains with different ploidy in the dataset presents a problem for phylogenetic analysis. In studies on diploid eukaryotes such as mammals, the standard approaches for constructing phylogenies of individuals from SNP data either exclude all heterozygous sites, or randomly choose one of the alleles at these sites (Lischer et al., 2014). In our preliminary analyses of the *K. marxianus* data, it was noticed that in the diploid strains, one allele was often very similar to the NBRC1777 reference sequence, but the other allele was considerably different. We were therefore motivated to construct a phylogenetic tree of the *K. marxianus* strains that kept the alleles separate—namely, a tree of haplotypes rather than a tree of strains.

**Figure 3:**
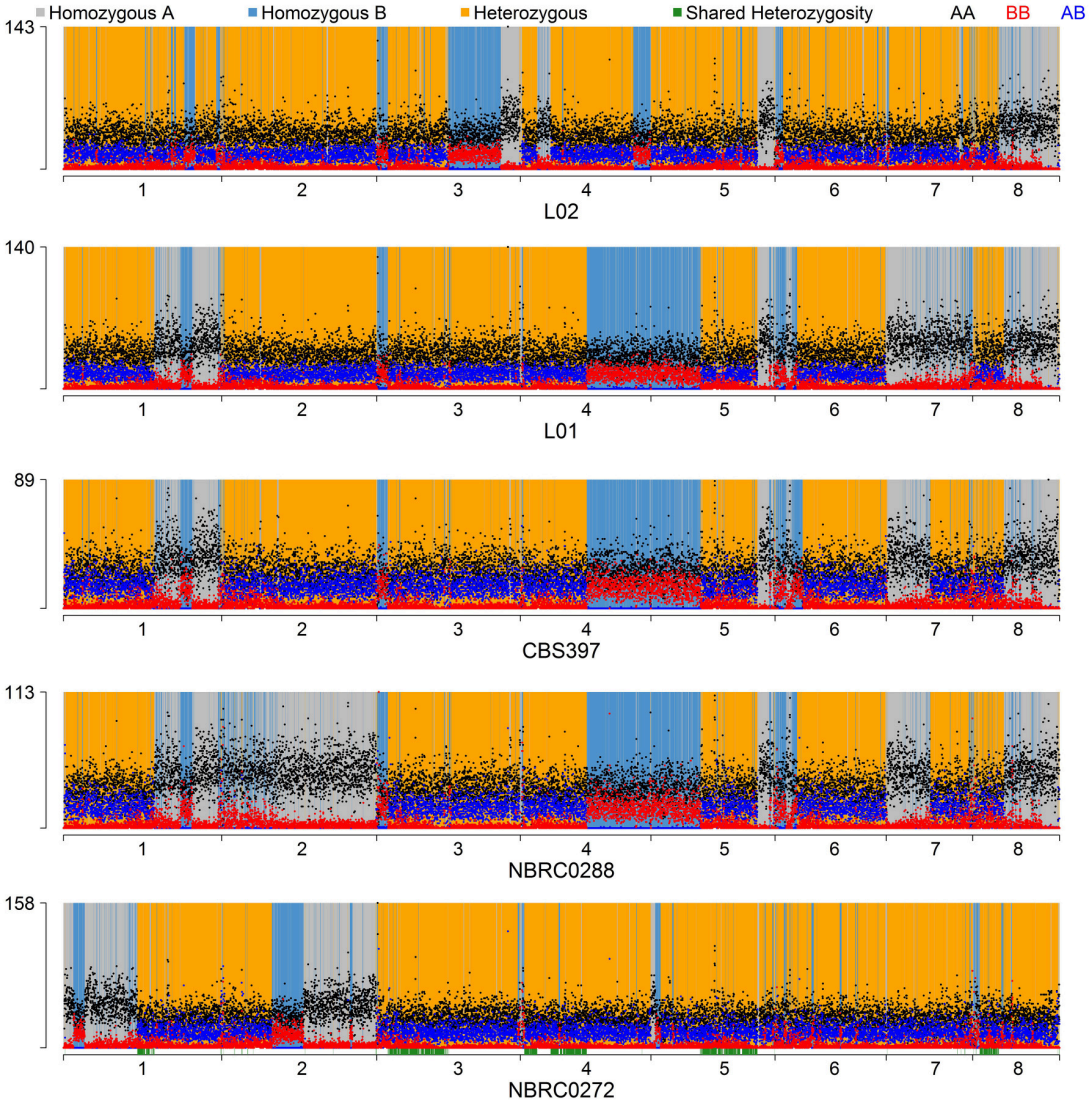
Haplotype assignment in diploid strains. For each 1-kb window through the genome, dots show the number of variable sites in the window with genotypes *AA* (black dots), *BB* (red dots), or *AB* (blue dots). Each window was then classified as either heterozygous (yellow background), homozygous for the *B* haplotype (blue background), or homozygous for the reference *A* haplotype (grey background) following rules as described in Methods and Figure S1. The green bars along the bottom axis show the regions (18%) that were heterozygous in all diploids and used for the haplotype phylogeny analysis.

**Figure 4:**
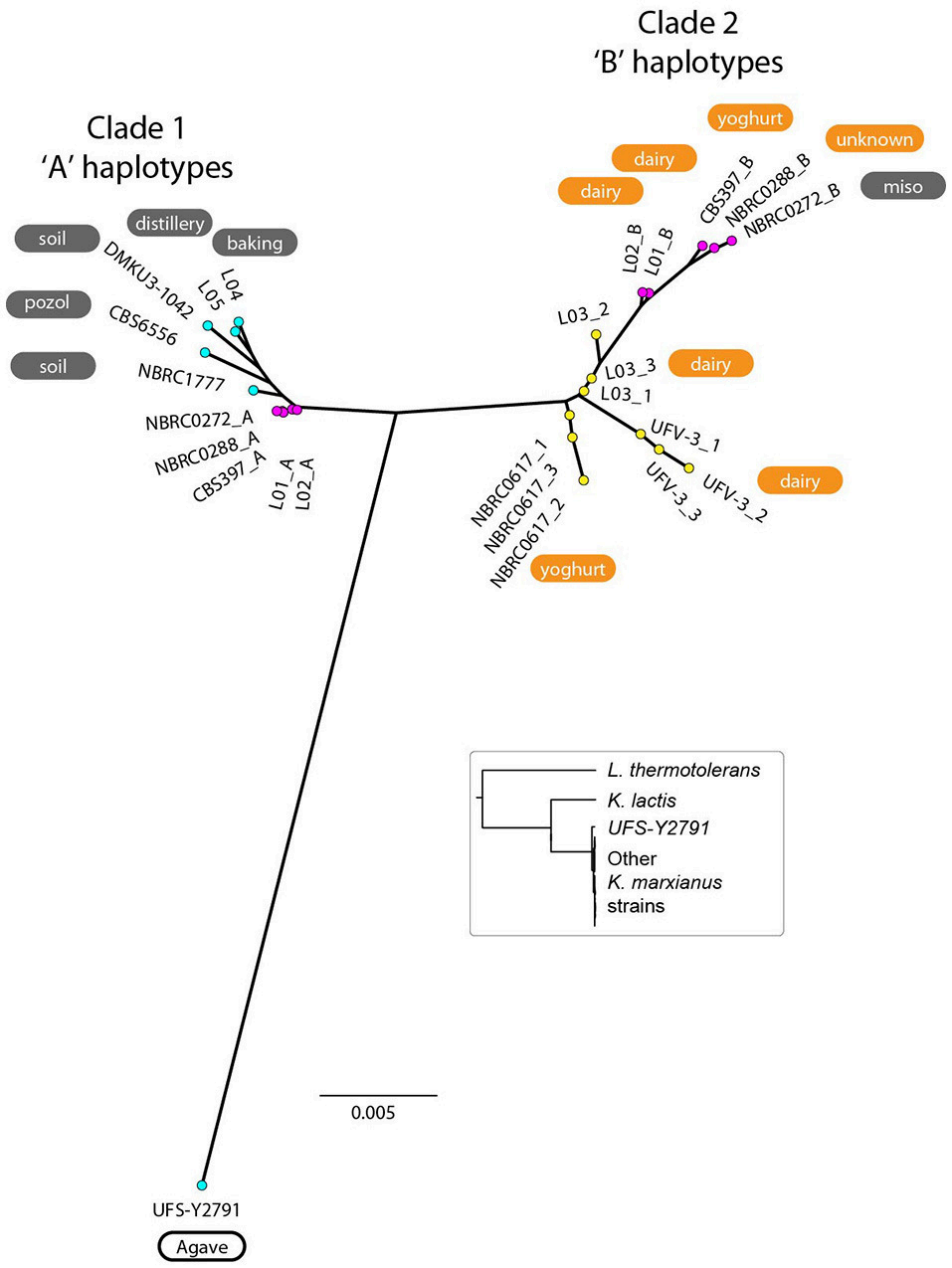
Phylogenetic tree of *K. marxianus* haplotypes. Coloured dots indicate the ploidy of strains as haploid (cyan; 1 haplotype per strain), diploid (magenta; 2 haplotypes per strain), or triploid (yellow; 3 haplotypes per strain). The environmental source of each strain is shown (for diploid strains, the source is only labeled on the “B” haplotype). Orange backgrounds indicate Lac+ strains, grey backgrounds indicate Lac− strains, and white backgrounds indicate Lac phenotypes not tested. The tree was constructed from SNP data from regions that are heterozygous in diploids, totalling 18% of the genome. Inset, phylogenetic tree constructed from DNA sequences of *GCN1* (an arbitrarily chosen large gene), confirming that UFS-Y2791 is a strain of *K. marxianus*.

To make a tree of haplotypes, we used a method previously developed to investigate the pathogenic yeast *Candida orthopsilosis*, which is an interspecies hybrid (Schröder et al., 2016). In each of the 5 diploid *K. marxianus* strains, each region of the genome was classified as either heterozygous, homozygous for the “A” haplotype (the haplotype more similar to the NBRC1777 reference), or homozygous for the “B” haplotype (the haplotype less similar to the reference) (Figure 3; see Methods for details). Approximately 18% of the genome was heterozygous in all 5 diploid strains, and we then extracted the sequences of the “A” and “B” haplotypes from these regions in each diploid. The aberrant (trisomic or tetrasomic) regions of the NBRC0272 genome were excluded from this dataset. A similar process was used to estimate the sequences of the three haplotypes present in each of the three triploid strains in these regions (see Materials and Methods). In the phylogenetic tree of haplotypes then generated, each haploid strain appears once, each diploid strain appears twice, and each triploid strain appears three times (Figure 4). Three clades are evident, but Clade 3 contains only the haploid strain UFS-Y2791. Despite the high divergence of UFS-Y2791 from the other strains, a phylogenetic tree using *K. lactis* and *Lachancea thermotolerans* as outgroups confirms that it is indeed a strain of *K. marxianus* (Figure 4, inset). All other *K. marxianus* haplotypes lie in Clades 1 and 2. Clade 1 contains all the haploid strains except the outlier UFS-Y2791, and the “A” haplotypes of each of the five diploid strains. Clade 2 contains the “B” haplotypes of the diploid strains, and all three haplotypes of the triploid strains.

### Lactose consumption phenotypes and *LAC12* genotypes

**Figure 5:**
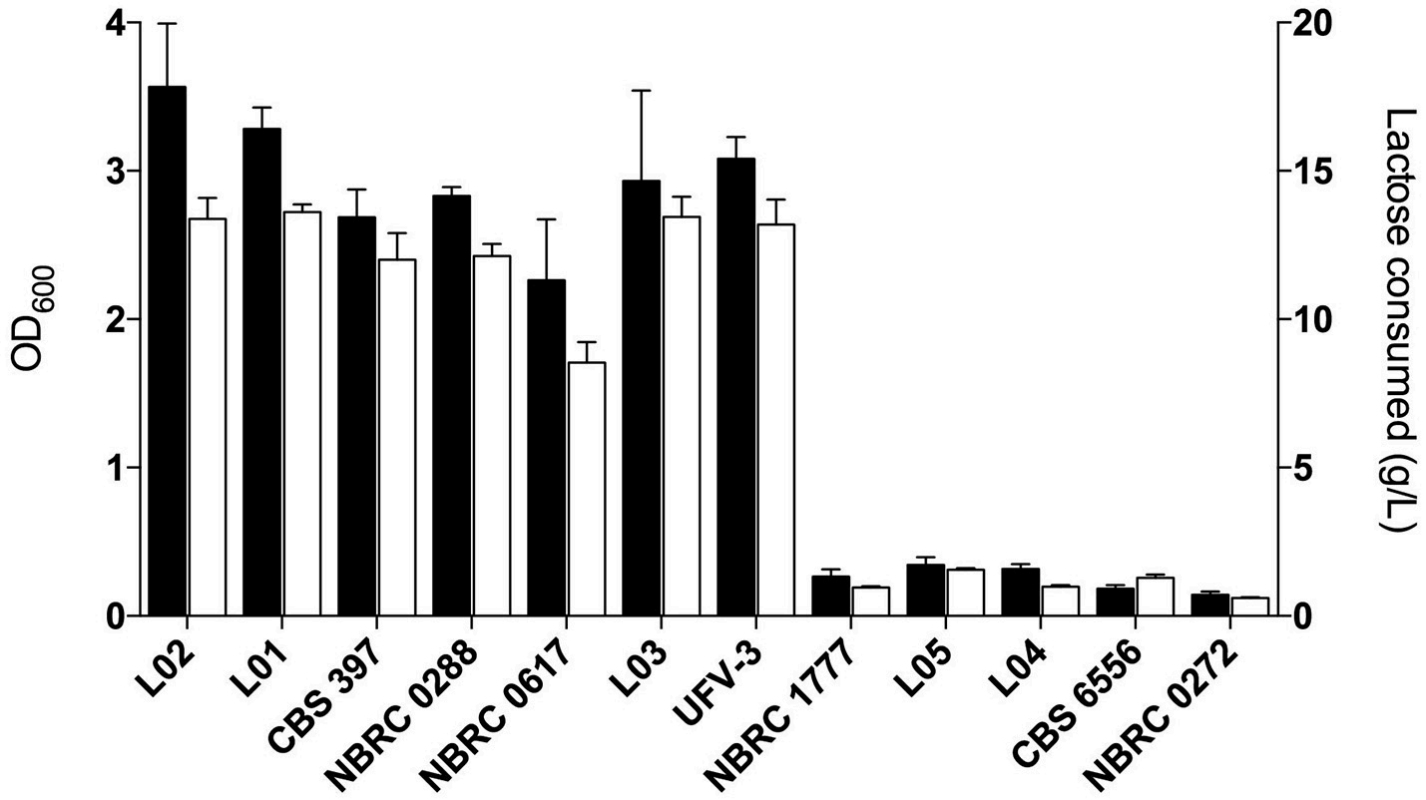
Growth and lactose consumption by *K. marxianus* strains. Growth in MM + 2 % lactose and lactose consumption are shown in black and white bars, respectively. Lactose consumption was calculated by subtracting the final and initial lactose concentrations in the medium.

When the environments from which the strains were isolated are considered, an unexpected relationship is apparent between environment, ploidy and clade (Figure 4). The six strains from “dairy” environments (and strain NBRC0288, from an unknown source) are all either diploid or triploid whereas, with the exception of strain NBRC0272 (isolated from miso), all the strains from “non-dairy” environments are haploid (Table 1). Since the use of lactose as a sugar source is considered important for growth of dairy yeasts, the capacity of the strains to grow on lactose was assessed to determine whether a similar pattern would emerge (Figure 5). Indeed, none of the tested haploid strains used lactose, whereas all the diploid and triploid strains were Lac+, again with the exception of NBRC0272, which is diploid but Lac−. Although DMKU3-1042 was not available for this study, our previous work has demonstrated that it is Lac− (Varela et al., 2017). We also previously established that the variable ability of *K. marxianus* strains to consume lactose is explained by polymorphism of a single gene, the lactose transporter *LAC12*, and that functional and non-functional (in terms of lactose transport) alleles of this gene differ by 13 key amino acid substitutions (Varela et al., 2017). BLAST searches against *de novo* assemblies of the genomes showed that the key polymorphisms in all the Lac+ strains in Figure 5 match were an exact match to the functional *LAC12*+ allele, except for NBRC0617 which matched in 11 positions. Similarly, all the Lac− strains exactly matched the non-functional *LAC12* allele, except for NBRC0272, which diverged at a single amino acid (Figure S4). None of the diploid strains is a *LAC12*+/− heterozygote, due to LOH at the left end of chromosome 3 where *LAC12* is located (the gene is only 15 kb from the telomere). Thus, although all five diploids are AB heterozygous for most of chromosome 3, the four Lac+ diploids are “BB” homozygous and the Lac− diploid strain NBRC0272 is “AA” homozygous in this region (Figure 3). In addition to the polymorphisms associated with a non-functional allele, the *LAC12* gene in NBRC0272 contains an internal stop codon (Figure S4).

## Discussion

### Ploidy in *K. marxianus* distinguishes dairy and non-dairy strains

Although this study relied on a relatively small set of strains (14), it delivered some remarkable insights into the life-cycle of *K. marxianus*. Our previous report that natural isolates of this yeast can be either haploid or diploid (Lane et al., 2011) was confirmed and then extended by the discovery of three triploid strains. The sample size is too small to draw statistical conclusions but it can be said that haploids, diploids, and triploids were present at roughly equal frequencies (43, 36, and 21% respectively) in this set. This appears to contrast with *K. lactis*, which is considered to be haploid, though it must be borne in mind that there are as yet no published production-level studies with that yeast. Variable ploidy is not uncommon in yeasts; for example, among 144 mainly clinical isolates of *S. cerevisiae*, the basal ploidy levels (i.e., ignoring aneuploid chromosomes) were haploid (11%), diploid (57%), triploid (16%), and tetraploid (16%) (Zhu et al., 2016).

The most striking finding was that all the isolates from a dairy environment were either diploid or triploid, whereas non-dairy isolates were haploid. Furthermore, it was possible to distinguish two genomic haplotypes, described here as “A” and “B” that mapped 13/14 strains into distinct clades (Clades 1 and 2). The 14th strain, UFS-Y2791 may represent a third clade. All the dairy isolates contained at least one of the B haplotype genomes, suggesting that this is a dairy-niche associated genome. In the case of the three triploid strains, the B genome was represented three times, whereas the diploid strains contained one A haplotype genome and one B haplotype genome. The sequence divergence (~2%) between the “A” and “B” haplotypes indicates that the diploid dairy strains were probably formed by mating between haploid representatives of Clades 1 and 2, as opposed to any other mechanism of ploidy change. Nevertheless, we were unable to examine *MAT* locus genotypes because the sequence assemblies are too fragmented in the *MAT/HML/HMR* regions. The phylogeny of the haplotypes indicates that the diploid dairy strains were formed by at least two independent matings between parents from the A and B clades, and that the A parents in these matings were very closely related to each other (much more so than the B parents). Triploids may have arisen by self-mating of B-haplotype (clade 2) strains, with one scenario being mating of a BB diploid with a B haploid to form a triploid; other routes to a triploid are also possible. It is implicit in these scenarios that B-haplotype haploid strains should also exist, though none were found in the current study. It is also notable that in our recent study developing an MLST method for *K. marxianus*, all 57 strains that were listed as coming from (6 different) dairy environments were heterozygous and therefore presumably diploid (Tittarelli et al., 2018). In fact, in that study, only 13/83 strains were homozygous in the regions included in the MLST. It is noted that one well-studied strain in the literature is *K. marxianus* CBS397 and this study and those of Fasoli et al. (2016) and Tittarelli et al. (2018) show that this strain is diploid (Fasoli et al., 2016; Tittarelli et al., 2018), which contrasts with what appears to be a previous erroneous suggestion based on long range PCR of the *MAT* locus that it was haploid (Lane et al., 2011).

The data suggest that the B-haplotype is a dairy-associated genome. It was gratifying, therefore, to identify one locus in this haplotype that confers a growth advantage in milk, the *LAC12* gene. Our previous work identified positions in the Lac12p where the functional protein had one particular amino acid and the non-functional protein a different one (Varela et al., 2017). In six of the strains with a B-haplotype genome, there was an exact match to this functional sequence and in the seventh (NBRC0617), there was a match in 11 positions (Figure S4). Since all these strains grew on lactose as a sole sugar source, this now allows us to propose that the number of amino acid positions that distinguish a functional and non-functional lactose-transporting Lac12p protein can be refined to these 11 amino acids though confirmation would require functional tests. The Lac− strain with the B-haplotype genome was NBRC0272, which is homozygous for the non-functional *LAC12* allele. This strain was isolated from a non-dairy environment (miso) and the most likely explanation is that it arose like the other diploids as a hybrid between an A and a B strain but since lactose transport was not required in its niche, it was possible for it to lose the B-haplotype *LAC12* allele through a LOH event at the left end of chromosome 3 whereas the other diploids lost the non-functional A-haplotype *LAC12* allele via a similar LOH event (Figure 4).

The situation in *K. marxianus* seems to resemble a pattern seen in *Saccharomyces* and *Zygosaccharomyces* species, where strains used in industrial processes or isolated from industrial environments are often polyploids or interspecies hybrids, whereas “natural” isolates (e.g., from non-anthropogenic environments) tend to be haploid or homozygous diploid (Hittinger, 2013; Suh et al., 2013; Wendland, 2014; Ortiz-Merino et al., 2017). This pattern is thought to reflect selection toward stress tolerance in the industrial environment, but toward maintenance of the ability to mate and sporulate in natural environments. If this is also the case with *K. marxianus*, it could be expected that the AB diploids display enhanced stress tolerance, at least over B-haplotype haploid strains in a dairy environment. Experiments to date have not succeeded in identifying any correlations between ploidy and stress tolerance (data not shown), but more studies that also include B-haplotype haploids are required to further address this question. As mentioned, B-haplotype strains have not yet been positively identified but there are Lac+ candidates worth investigating, for example, *K. marxianus* NCYC1424, shown to be homozygous by Tittarelli et al. (2018), and *K. marxianus* NCYC1429, which appears to be haploid based on genetic crossing (Varela et al., 2017).

### Sequence diversity, aneuploidy, and LOH in *K. marxianus*

One of the aims of this study was to assess if the wide phenotypic diversity that has been observed in *K. marxianus* was reflected in its genome diversity. The large number of SNPs (>500 k) observed in the set of *K. marxianus* strains used in our study shows relatively high SNP diversity. We found an average pairwise difference (π) of 12 × 10^−3^, which is comparable with reported values from other yeasts; for example, 4 × 10^−3^ in *S. cerevisiae*, 12 × 10^−3^ in *S. uvarum*, and 17 × 10^−3^ in *L. kluyveri* (Peter and Schacherer, 2016).

Previous studies with *S. cerevisiae* isolates showed variable ploidy (from 1 to 4 copies of the genome), aneuploidy (unequal copy numbers of different chromosomes), or variation in the copy number of segments of chromosomes (Hose et al., 2015; Strope et al., 2015; Zhu et al., 2016). Similar to *S. cerevisiae* but unlike *L. kluyveri* (Friedrich et al., 2015), all these phenomena were observed in this study of *K. marxianus*. The most unambiguous example of aneuploidy in *K. marxianus* is the presence of an extra copy of chromosome 7 in NBRC0617, but, as described in the results, there are multiple other likely cases of aneuploidies or copy number variation that would need to be investigated in more detail.

There are also quite extensive regions of LOH in the diploid strains (25–51% by genome length) but not in the triploids. LOH arises when the genome homogenises in a region and it is expected to be rarer in triploid strains, because it will only be apparent if all three copies of a genomic region were homogenised. The shared patterns of LOH in different diploid *K. marxianus* isolates was unexpected. This observation could indicate that these strains are closely related and are mitotic descendants of a recent diploid common ancestor that had already lost heterozygosity in the shared regions. Alternatively, it could indicate that this species mostly reproduces by mitosis and rarely goes through meiosis and sporulation, at least in the dairy environment. Nonetheless, the divergence between the *K. marxianus* clades is low enough that it would not be expected to cause problems in meiosis. In *S. paradoxus*, crosses between strains with sequence divergence of up to 4.6% can still produce viable gametes, as long as the genomes are collinear (Liti et al., 2006). It should also be considered that dairy is probably not the original niche for *K. marxianus* and the strains that we studied were most likely selected during a fermentation process. This could have specifically selected hybrids between A and B clade strains, and may also promote LOH. Other than the preservation of the functional lactose transporting allele of *LAC12* (B-haplotype), there was not an obvious preference for either the A-haplotype or the B-haplotype during LOH events (Figure 3). Because lactose utilisation confers a benefit during growth in milk, one can speculate that the LOH of the region containing the functional *LAC12* gene is an adaptive response. It is not possible to say, however, whether or not other selective pressures played a role in determining the overall patterns of LOH.

### Implications for biotechnology

This study focused on a small set of strains of biotechnological interest and therefore may not be fully representative of the species diversity. Indeed, one strain (UFS-Y2791) was far more diverse than the others, suggesting that there is further diversity to be accessed. Given that UFS-Y2791 was isolated from agave juice (in South Africa), it will be interesting to see whether strains associated with tequila/mezcal fermentation (also from agave) in Mexico show any relationship to this strain. The divergence between strains used, either deliberately or traditionally, in the food biotechnology sector is very significant in comparison to those isolated from “natural” environments. Perhaps the natural state of *K. marxianus* is haploid like its sister *K. lactis*, and diploids only arise after biotechnological selection. It is possible that diploids will have advantages, though, other than for lactose utilisation, these are not yet apparent. Haploid strains are much easier to engineer and manipulate so, for most biotechnological applications, it may be preferable to choose Clade 1 (A haplotype) strains. Nonetheless, divergent alleles in Clade 2 (B haplotype) may also be functionally important (for example *LAC12*) so this will still need to be considered in future studies.

## Author contributions

RO-M and JV: contributed equally to the paper, they carried out the bulk of the experimental and bioinformatic analysis and wrote the manuscript; AC: contributed with strain handling and sample preparation for DNA sequencing; CW, NK, J-MG, WdS, and HH: sequenced *K. marxianus* strains for the study; KW and JM: conceived the study, supervised the research, analysed and interpreted data and contributed to writing the manuscript.

### Conflict of interest statement

CW was employed by company Lallemand Inc and J-MG and NK by Heineken. The other authors declare that the research was conducted in the absence of any commercial or financial relationships that could be construed as a potential conflict of interest.

## Supplementary material

Figure S1 Method of classifying 1-kb genomic windows into haplotypes. The cutoff values chosen are marked by triangles in all panels (windows with ≥3 *AB* SNPs were classified as *AB* heterozygous windows; windows with ≥9 *BB* SNPs were classified as *BB* heterozygous windows; other windows were classified as *AA* homozygous windows). The data plotted are for the diploid strain L01. **(A)** Histogram of numbers of SNPs of each type, in all 1-kb windows in the genome. **(B)** Heatmap showing the distribution of numbers of *AB* and *BB* SNPs per window. Cells in the matrix show numbers of windows. The cutoff values were chosen to coincide with minima on the two axes. **(C)** Distribution of numbers of *BB* SNPs in windows that have zero *AB* SNPs.

Figure S2 Allele frequencies and sequence coverage in three strains on **(A)** chromosome 7 and **(B)** chromosome 2. The *left* and *middle panels* show distributions of allele frequency on each chromosome, as in Figure 1. The centromere is marked by a vertical red line. The *right panels* show log_2_ ratios between observed and expected sequence coverage, as in Figure 2. Cyan lines for NBRC0617 indicate the value expected for the 1.33-fold increase in coverage that would result from a fourth copy of a region in a triploid. The vertical orange lines in **(B)** mark the 150 kb region from with increased coverage in NBRC0617 (coordinates 420–570 kb).

Figure S3 Allele frequencies on each chromosome of NBRC0272. Details are as in Figures 1A,B. In the plots on the right, red vertical lines mark centromeres and blue lines mark the rDNA array.

Figure S4 Multiple alignment of Lac12p sequences from the strains used in this study. Amino acid sequences shown were derived from the haplotypes of each strain. Haplotypes from diploid and triploid strains are denoted by letters and numbers, respectively. The residues marked in color are those associated with either functional or non-functional alleles. The positions marked in blue were previously part of the set that distinguished alleles but these are not conserved in all haplotype B Lac12p. Those in red are those that still distinguish based on functionality and an additional differentiating AA at position 475 (pink shading) that was overlooked in the previous study is also marked. Thus, based on sequences currently available, there are 11 differentiating amino acids. The stop codon in the NBRC0272_B sequence at position 139 is indicated with a green asterisk.

Table S1 SNP density.
