# Supplementary figures and images for: Ploidy Variation in Kluyveromyces marxianus Separates Dairy and Non-dairy Isolates
Source: Front Genet. 2018 Mar 21;9:94. doi: 10.3389/fgene.2018.00094 (PMC5871668; doi:10.3389/fgene.2018.00094)

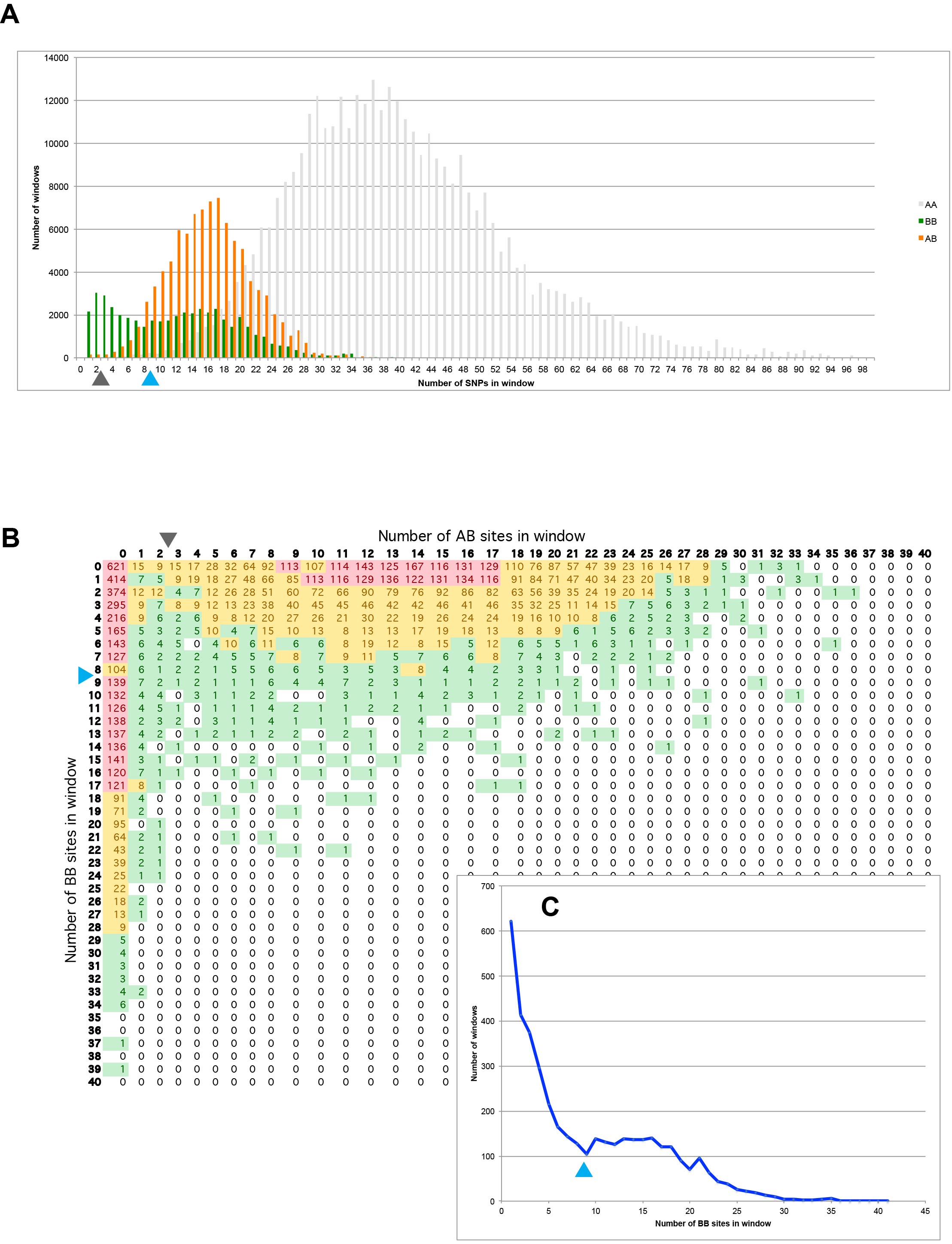

Supplement: Figure S1 — Method of classifying 1-kb genomic windows into haplotypes. The cutoff values chosen are marked by triangles in all panels (windows with ≥3 AB SNPs were classified as AB heterozygous windows; windows with ≥9 BB SNPs were classified as BB heterozygous windows; other windows were classified as AA homozygous windows). The data plotted are for the diploid strain L01. (A) Histogram of numbers of SNPs of each type, in all 1-kb windows in the genome. (B) Heatmap showing the distribution of numbers of AB and BB SNPs per window. Cells in the matrix show numbers of windows. The cutoff values were chosen to coincide with minima on the two axes. (C) Distribution of numbers of BB SNPs in windows that have zero AB SNPs. [file Image1.JPEG]

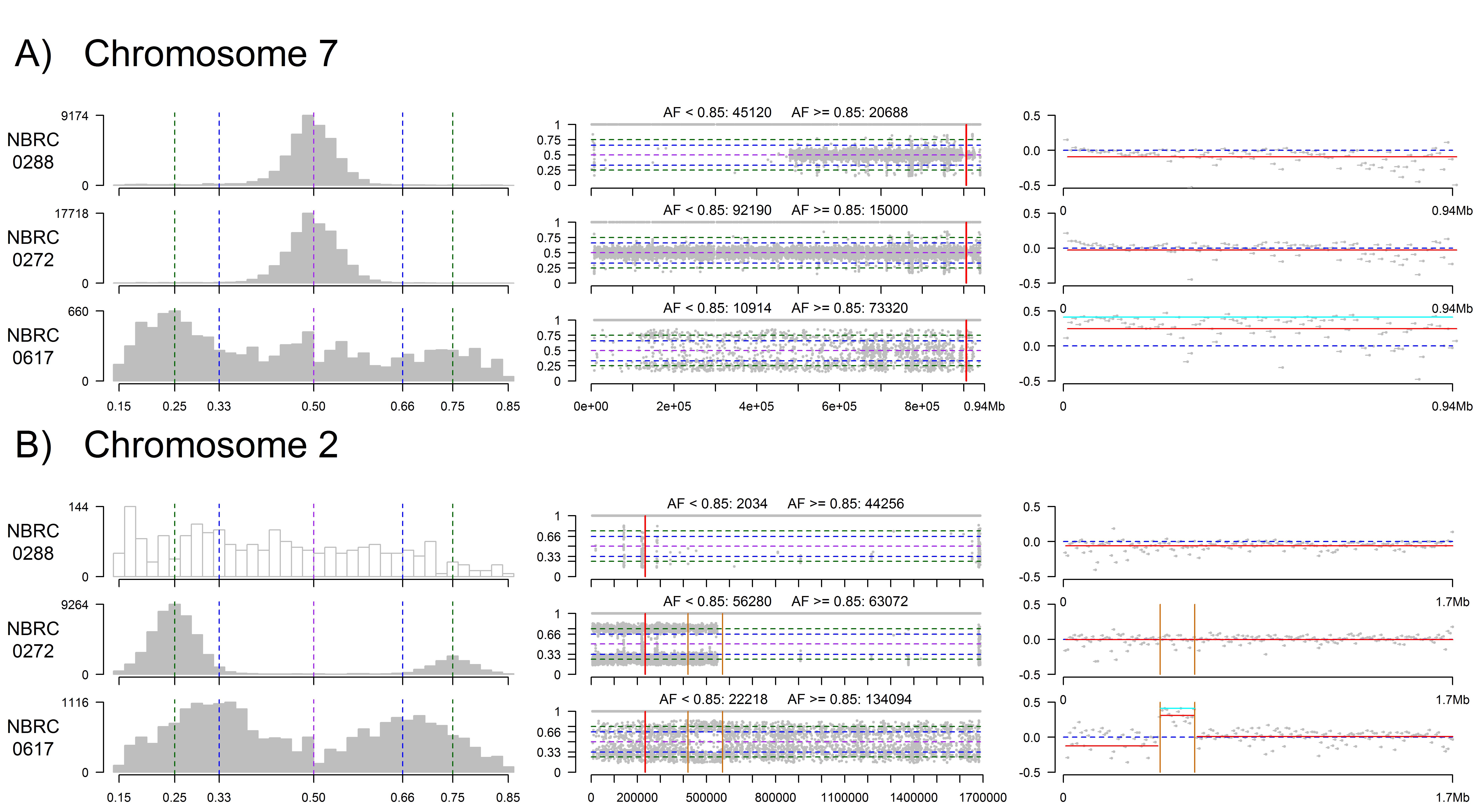

Supplement: Figure S2 — Allele frequencies and sequence coverage in three strains on (A) chromosome 7 and (B) chromosome 2. The left and middle panels show distributions of allele frequency on each chromosome, as in Figure 1. The centromere is marked by a vertical red line. The right panels show log2 ratios between observed and expected sequence coverage, as in Figure 2. Cyan lines for NBRC0617 indicate the value expected for the 1.33-fold increase in coverage that would result from a fourth copy of a region in a triploid. The vertical orange lines in (B) mark the 150 kb region from with increased coverage in NBRC0617 (coordinates 420–570 kb). [file Image2.JPEG]

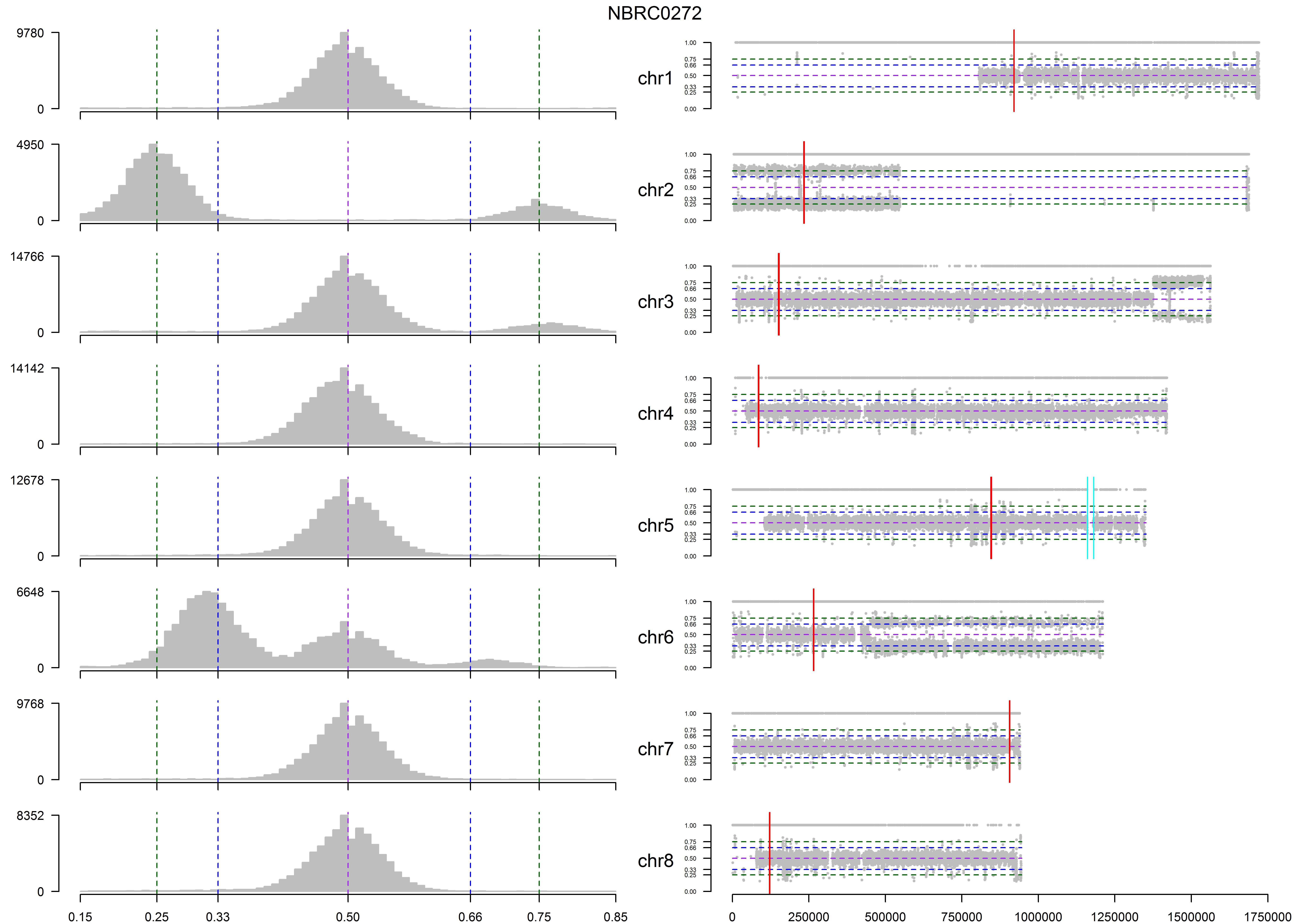

Supplement: Figure S3 — Allele frequencies on each chromosome of NBRC0272. Details are as in Figures 1A,B. In the plots on the right, red vertical lines mark centromeres and blue lines mark the rDNA array. [file Image3.JPEG]

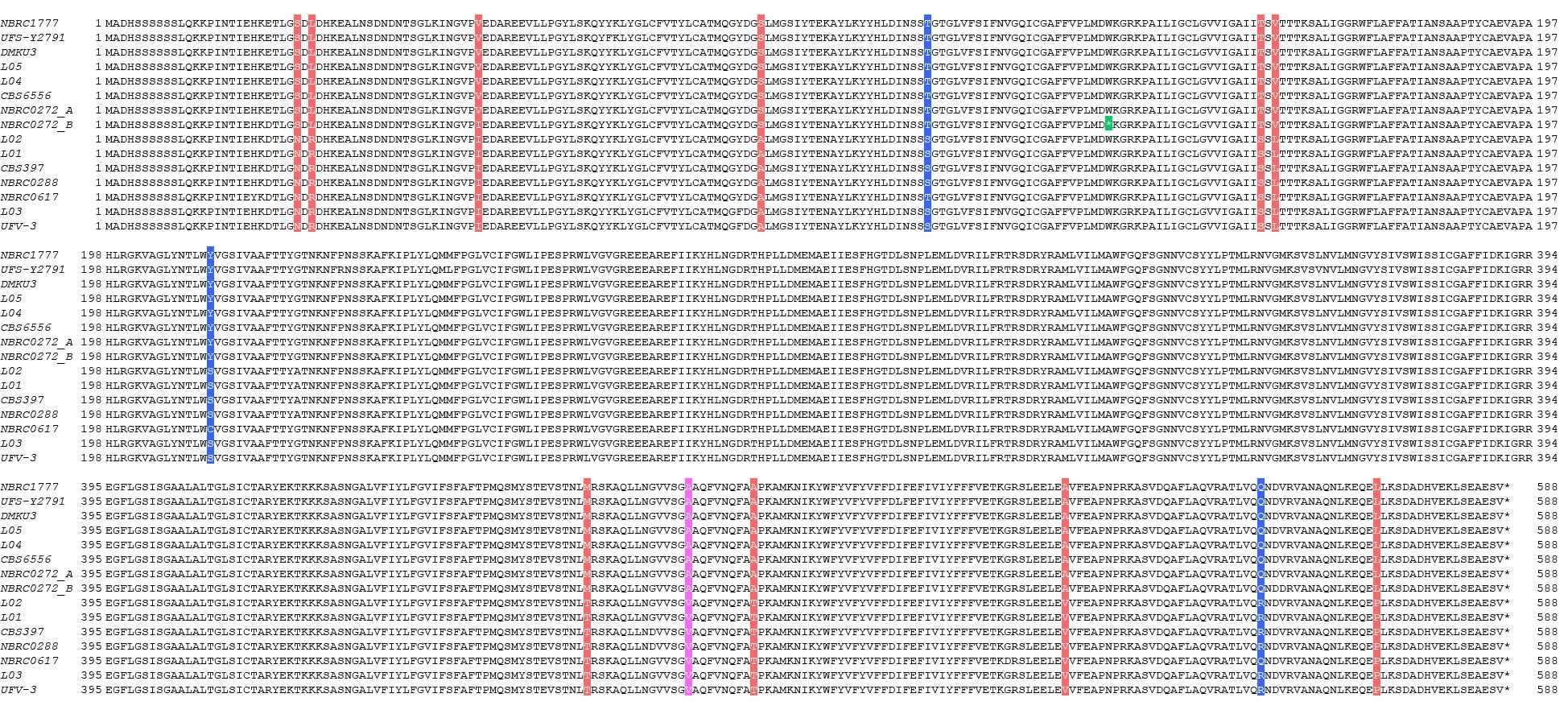

Supplement: Figure S4 — Multiple alignment of Lac12p sequences from the strains used in this study. Amino acid sequences shown were derived from the haplotypes of each strain. Haplotypes from diploid and triploid strains are denoted by letters and numbers, respectively. The residues marked in color are those associated with either functional or non-functional alleles. The positions marked in blue were previously part of the set that distinguished alleles but these are not conserved in all haplotype B Lac12p. Those in red are those that still distinguish based on functionality and an additional differentiating AA at position 475 (pink shading) that was overlooked in the previous study is also marked. Thus, based on sequences currently available, there are 11 differentiating amino acids. The stop codon in the NBRC0272_B sequence at position 139 is indicated with a green asterisk. [file Image4.JPEG]
